# *Montanoa frutescens* and *Montanoa grandiflora* Extracts Reduce Anxiety-Like Behavior during the Metestrus-Diestrus Phase of the Ovarian Cycle in Wistar Rats

**DOI:** 10.1155/2014/938060

**Published:** 2014-04-01

**Authors:** Juan Francisco Rodríguez-Landa, Julio Vicente-Serna, Luis Alfredo Rodríguez-Blanco, María de Jesús Rovirosa-Hernández, Francisco García-Orduña, Miguel Carro-Juárez

**Affiliations:** ^1^Instituto de Neuroetología, Universidad Veracruzana, Avenida Dr. Luis Castelazo s/n, Colonia Industrial Ánimas, 91001 Xalapa, VER, Mexico; ^2^Facultad de Química Farmacéutica Biológica, Universidad Veracruzana, 91190 Xalapa, VER, Mexico; ^3^Programa de Tutoría para la Investigación, Universidad Veracruzana, 91001 Xalapa, VER, Mexico; ^4^Laboratorio de Comportamiento Reproductivo, Escuela de Medicina Veterinaria y Zootecnia, Universidad Autónoma de Tlaxcala, 90000 Tlaxcala, TLAX, Mexico

## Abstract

In previous studies, the anxiolytic-like effects of *Montanoa tomentosa* and *Montanoa frutescens* were reported in male rats, but the potential anxiolytic-like effects of *Montanoa* plants during the different phases of the ovarian cycle in rats remain to be explored. The anxiolytic-like effects of the aqueous crude extracts of *M. frutescens* (25 and 50 mg/kg) and *M. grandiflora* (25 and 50 mg/kg) in the elevated plus maze were investigated in Wistar rats during the estrous cycle and compared with 2 mg/kg diazepam as a reference anxiolytic drug. To investigate any motor effect (i.e., hyperactivity, no changes, or hypoactivity) associated with the treatments, the rats were evaluated in the open field test. The *M. frutescens* (25 and 50 mg/kg) and *M. grandiflora* (50 mg/kg) extracts exerted anxiolytic-like effects during the metestrus-diestrus phase, similar to diazepam, without disrupting spontaneous motor activity. No significant effects of the extracts were detected in either behavioral test during the proestrus-estrus phase, whereas diazepam produced motor hypoactivity in the open field test. These results indicate that the *M. frutescens* and *M. grandiflora* extracts possess anxiolytic-like effects that depend on the ovarian cycle phase, supporting the Mexican ancient medicinal use of these plants to ameliorate anxiety disorders.

## 1. Introduction

Generalized anxiety disorder is one of the most common psychiatric disorders, affecting a high percentage of the general population around the world [1]. Anxiety disorders possess marked gender differences and occur more often in women than in men [2–5]. The reproductive cycle of women exhibits fluctuations in plasma and brain steroid hormone concentrations (e.g., estradiol and progesterone, among others) that are related to anxiety and mood swings. Low concentrations of steroid hormones are associated with irritability, anxiety, or depressive symptoms and occur during the premenstrual, postpartum, and climacteric periods [6–8]. Interestingly, rats exhibit a predisposition to display more anxiety- and depressive-like states when lower concentrations of ovarian hormones are observed during the metestrus-diestrus phase of the ovarian cycle. During the ovarian cycle phases (i.e., proestrus-estrus phase) when high concentrations of steroid hormones are observed, anxiety- and depressive-like behaviors diminish [9, 10].

Variations in ovarian hormone levels across the estrous cycle in female rats permit the evaluation of anxiety- and depressive-like behaviors under the presence of standard anxiolytic drugs [11–13] and plant extracts [14] with anxiolytic or antidepressant activity. This has permitted investigators to explore potential therapeutic regimens to ameliorate anxiety- or depressive-like behaviors associated with naturally occurring low plasma concentrations of steroid hormones.

In ancient Mexican traditional medicine, some medicinal plants have been reported to possess anxiolytic effects and are currently recommended for the treatment of “anxious states.” The* Badianus Codex* or* Libellus de Medicinalibus Indorum Herbis* written in 1552 [15] describes the use of* Cihuapatli* (“women's medicine” in the Nahuatl language) for the treatment of mood and “nervous disorders,” an anxiety-like behavior that is mentioned in the context of ancient Mexican culture. Early reports by Ximenez (1615) stated, “Cihuapatli resolves the mood changes and nerves in an admirable form” [16]. Cihuapatli is the common name assigned to some plants included in the genus* Montanoa* (family: Asteraceae; tribe: Heliantheae), in which* M. tomentosa*,* M. frutescens*, and* M. grandiflora *are included. The aqueous crude extract from these plants has been used for centuries in traditional Mexican medicine as a remedy for reproductive impairments, anxiety, and mood disorders [16–19]. Recently, the aqueous extracts of* M. tomentosa* [20] and* M. frutescens* [21] were reported to produce anxiolytic-like effects that are similar to diazepam in Wistar rats, and the participation of *γ*-aminobutyric acid-A (GABA_A_) receptors in their anxiolytic-like effect was identified. Nonetheless, although some anxiolytic-like effects of* M. frutescens* extract have been corroborated in male rats, the anxiolytic-like effects of* M. frutescens* and* M. grandiflora* in the female genus remain unexplored. We hypothesized that* M. frutescens* and* M. grandiflora* extracts reduce anxiety-like behavior during the metestrus-diestrus phase of the estrous cycle in rats. To test this hypothesis, we used the elevated plus maze, a well-accepted model that tests clinically effective anxiolytic drugs [22, 23], to evaluate the effects of several doses of* M. frutescens* and* M. grandiflora* aqueous crude extracts on anxiety-like behavior during different phases of the ovarian cycle in Wistar rats. We also made comparisons with an anxiolytic dose of diazepam.

## 2. Material and Methods

### 2.1. Animals

Female Wistar rats were obtained from a local strain supplied by Harlan (Mexico City, Mexico). Adult female rats, weighing 200–250 g, were included in the experiments. The rats were housed in Plexiglas cages (six to seven rats per cage; 44 cm width, 33 cm length, and 20 cm height) under a 12 h/12 h light/dark cycle (lights on at 7:00 a.m.) at an average temperature of 25°C (±2°C) with* ad libitum* access to purified water and food (Harlan, Mexico). All of the experimental procedures were performed according to the Guide for the Care and Use of Laboratory Animals published by the National Institutes of Health [24] and Norma Oficial Mexicana para el Cuidado y Uso de Animales de Laboratorio [25]. The general protocol received authorization (MVZ-189/12) from the Comité Interno de Ética de la Escuela de Medicina Veterinaria y Zootecnia de la Universidad Autónoma de Tlaxcala.

### 2.2. Preparation of* M. frutescens* and* M. grandiflora* Extracts

*M. frutescens *and* M. grandiflora* were collected in their natural habitat in the state of Tlaxcala, Mexico, in September 2011 and were authenticated by a specialist from the Herbarium of the* Universidad Autónoma de Tlaxcala*, where voucher specimens are preserved and the plants are cultivated (serial number of* M. frutescens* UATX11 and* M. grandiflora* UATX12). The doses of the aqueous crude extracts of* M. frutescens *and* M. grandiflora* used in the present study are equivalent to those used for humans and were prepared according to previous studies [21], in which 25 and 50 mg/kg but not higher doses of* M. frutescens* produce anxiolytic-like effects. Briefly, the leaves of* M. frutescens *and* M. grandiflora* were collected and prepared for drying for 20 days. Once dried, the material was ground into a fine powder (average 1 g), which was mixed with 20 mL of purified water. This mixture was warmed for approximately 10 min, just before boiling. The obtained infusion was filtered and oven-dried at a temperature of 55°C, and the brownish residue of the extract yield was calculated as 80 mg. The dried extract of the plant was maintained at 3°C and then used to prepare the stock solutions. In the present study, a 50 mg/mL solution was initially prepared and then diluted to obtain equivalent solutions of 25 mg/mL. Considering that in traditional Mexican medicine similar effects are attributed to* M. frutescens* and* M. grandiflora* extracts [17, 18] we also tested the effects of 25 and 50 mg/kg of the* M. grandiflora* extract on anxiety-like behavior in female rats in the present study. The extracts that corresponded to each dose were prepared 40 min prior to administration to avoid modifications in the chemical properties of the extracts.

### 2.3. Behavioral Tests

To evaluate the effects of the treatments, the same rats were evaluated in the elevated plus maze (5 min) and subsequently in the open field (5 min) as previously reported [20, 21].

#### 2.3.1. Elevated Plus Maze

The apparatus was constructed of wood and situated in a brightly lit room (40 lux). The apparatus consisted of two opposite open and closed arms set in a plus configuration. The dimensions of the open arms were 50 cm length × 10 cm width, and the closed arms were 50 cm length × 10 cm width × 40 cm height. The entire maze was elevated 50 cm above the floor. A digital video camera (Sony, DCR-SR42, 40x optical zoom, Carl Zeiss lens) was installed above the apparatus to record the rats' activity. Later, two independent observers measured the behavioral variables until reaching at least 95% agreement in the measurements.

To evaluate the effects of the treatments, the rats were placed in the center of the maze, facing an open arm. The evaluated variables were (i) the time spent in the open arms, (ii) the number of entries into the open arms, (iii) the total number of entries (open arms + closed arms), and (iv) the percentage of open arm entries ((open entries)/(total entries) × 100). These variables were selected based on previous studies, in which these measures were shown to be reliable indicators of experimental anxiety [26, 27]. Rats that fell from the apparatus were discarded from subsequent data analysis.

#### 2.3.2. Open Field Test

To evaluate the effects of the* M. frutescens *and* M. grandiflora* extracts and diazepam on spontaneous motor activity, the rats were individually subjected to a 5 min open field test. The open field apparatus was an opaque Plexiglas cage (44 × 33 cm) with 20 cm high walls. The floor was delineated into 12 squares (11 × 11 cm). A digital video camera (Sony, DCR-SR42, 40x optical zoom, Carl Zeiss lens) was installed above the cage to record the rats' activity. Later, two independent observers measured the behavioral variables. General motor activity was examined to determine whether the treatments caused hypoactivity, hyperactivity, or no motor changes, which could interfere with behavioral activity in the elevated plus maze. At the onset of the test, the rats were gently placed in one of the corners of the cage, and the following variables were measured: (i) the number of squares crossed by the rat (i.e., when an animal passed from one square to another with its rear legs), (ii) the time in seconds spent in rearing (i.e., when the rat acquired a vertical posture with respect to the cage floor), (iii) the time in seconds spent in grooming (i.e., paw licking, nose/face grooming (strokes along the snout), head washing (semicircular movements over the top of the head and behind the ears), body grooming/scratching (body fur licking and scratching the body with the hind paws), leg licking, and tail/genital grooming (licking of the genital area and tail)) [28, 29], and (iv) total resting time in seconds (i.e., when the rat acquired a quiet posture on the floor without movements). After each test session, the elevated plus maze apparatus and open field cage were carefully cleaned with a 10% ethanol solution to remove the scents of previously evaluated animals. Five minutes elapsed between each test to allow the odors and cleaning solutions to dissipate.

### 2.4. Vaginal Smears

Before initiating the behavioral tests, we obtained daily vaginal smears from the rats. Only females with three continuous regular cycles (4-5 days) were included in the study. The vaginal samples were gently collected by inserting the tip of a medicine dropper into the vagina, flushing saline in and out, and placing the fluid onto microscope slides. The estrous cycle stage was determined immediately by optical microscopy (40x magnification). The smears were classified by estimating the relative proportions of leukocytes, nucleated epithelial cells, and cornified epithelial cells [30] as follows: diestrus (predominance of large numbers of leukocytes, some nucleated and almost no cornified cells), proestrus (primarily large, round, nucleated cells), estrus (many cornified cells), and metestrus (large numbers of leukocytes, some cornified cells, and almost no nucleated cells). We classified the animals into two subgroups for the statistical analysis (i.e., proestrus-estrus and metestrus-diestrus) by considering previous studies in which proestrus-estrus was characterized by low anxiety-like indicators associated with high concentrations of ovarian hormones and metestrus-diestrus was characterized by high anxiety-like indicators associated with low concentrations of ovarian hormones [10, 11, 31].

### 2.5. Experimental Groups and Treatments

A total of 104 rats were included in the study and assigned to six independent groups. The control group (vehicle; *n* = 17) received purified water (1 mL/kg), which was used to prepare the plant extracts. Two groups received 25 mg/kg/mL (*n* = 16) and 50 mg/kg/mL (*n* = 17) of the aqueous extract of* M. frutescens*. Two groups received 25 mg/kg/mL (*n* = 18) and 50 mg/kg/mL (*n* = 19) of the aqueous extract of* M. grandiflora*. All of the doses of the extracts were administered orally by gavage. Purified water was used as the vehicle, considering that it is used in ancient traditional medicine to prepare* Montanoa* extracts [15, 16]. An additional group received 2 mg/kg diazepam, i.p. (*n* = 17), as a reference anxiolytic drug to validate the experimental conditions of the elevated plus maze and make comparisons with the effects of the* Montanoa *extracts. The 2 mg/kg dose of diazepam was selected according to previous studies that reported anxiolytic-like effects of this dose in the elevated plus maze [27, 32]. Diazepam was acquired from Laboratorios Cryopharma, DF, Mexico. All of the treatments were administered in a single dosage in an equivalent volume of 1 mL/kg. Thirty minutes after the corresponding treatment, the rats were evaluated in the elevated plus maze (5 min) and subsequently in the open field (5 min). After the open field test, vaginal smears were obtained to assign each rat from each treatment group to its corresponding subgroup (i.e., proestrus-estrus and metestrus-diestrus groups).

### 2.6. Statistical Analysis

All of the data were analyzed using two-way analysis of variance (ANOVA) with independent groups, with treatment as the first factor and phase of the ovarian cycle as the second factor. Values of *P* ≤ 0.05 in the ANOVA were followed by the Student-Newman-Keuls* post hoc* test. The data are expressed as mean ± standard error.

## 3. Results

### 3.1. Vaginal Smears

The microscopic analysis of vaginal smears permitted us to identify the phase of the ovarian cycle in each rat to be included in each subgroup (i.e., proestrus-estrus and metestrus-diestrus phases) for the statistical analysis. The number of animals in each subgroup was the following: proestrus-estrus (*n* = 8-9 per group) and metestrus-diestrus (*n* = 8−10 per group).

### 3.2. Elevated Plus Maze

The two-way ANOVA showed significant differences in the time spent in the open arms according to the phase of the ovarian cycle (*F*_1,92_ = 16.85, *P* < 0.001) and treatment (*F*_5,92_ = 10.95, *P* < 0.001). The interaction between factors was also significant (*F*_5,92_ = 8.74, *P* < 0.001). The* post hoc* test showed that, similar to diazepam, rats treated with 25 and 50 mg/kg of the* M. frutescens* extract and 50 mg/kg of the* M. grandiflora* extracts during the metestrus-diestrus phase spent more time in the open arms compared with rats in the metestrus-diestrus phase in the control group. No other effects of the treatments on the time spent in the open arms during the proestrus-estrus phase were detected.

**Figure 1:**
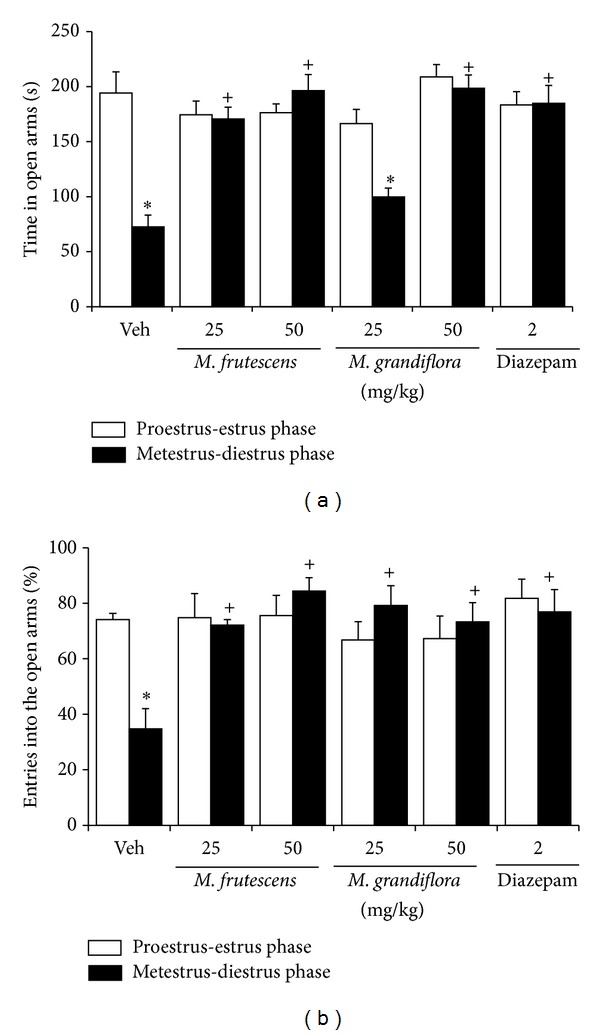
Elevated plus maze. (a) Time spent in the open arms and (b) percentage of entries into the open arms during the 5 min test. *P < 0.05, compared with proestrus-estrus phase in the same group; ^+^ *P* < 0.05, compared with metestrus-diestrus phase in the control group (Veh) (two-way ANOVA followed by Student-Newman-Keuls* post hoc* test).

The two-way ANOVA of the percentage of entries into the open arms did not reveal statistically significant differences considering the phase of the ovarian cycle (*F*_1,92_ = 0.64, *P* = 0.425), but statistically significant differences were detected among treatments (*F*_5,92_ = 3.44, *P* < 0.007). The* post hoc* test showed that 50 mg/kg of the* M. frutescens* extract and 2 mg/kg diazepam increased this variable compared with the control group. The interaction between factors was also significant (*F*_5,92_ = 3.59, *P* < 0.005). The* post hoc* test showed that, similar to diazepam, rats treated with 25 and 50 mg/kg of the* M. frutescens *and* M. grandiflora *extracts during the metestrus-diestrus phase exhibited an increase in the percentage of entries into the open arms compared with the control group in the metestrus-diestrus phase. No other effects of the treatments on this variable were detected during the proestrus-estrus phase (Figure 1(b)).

The analysis of the number of entries into the open arms did not reveal statistically significant differences between phases of the ovarian cycle (*F*_1,92_ = 0.68, *P* = 0.413) or among treatments (*F*_5,92_ = 0.54, *P* = 0.748) or an interaction between factors (*F*_5,92_ = 1.64, *P* = 0.159). The analysis of the total number of entries into the arms (open arms + closed arms) did not reveal significant differences between phases of the ovarian cycle (*F*_1,92_ = 0.01, *P* = 0.95) or treatments (*F*_5,92_ = 2.08, *P* = 0.074) or an interaction among factors (*F*_5,92_ = 0.60, *P* = 0.703; Table 1).

### 3.3. Open Field Test

**Figure 2:**
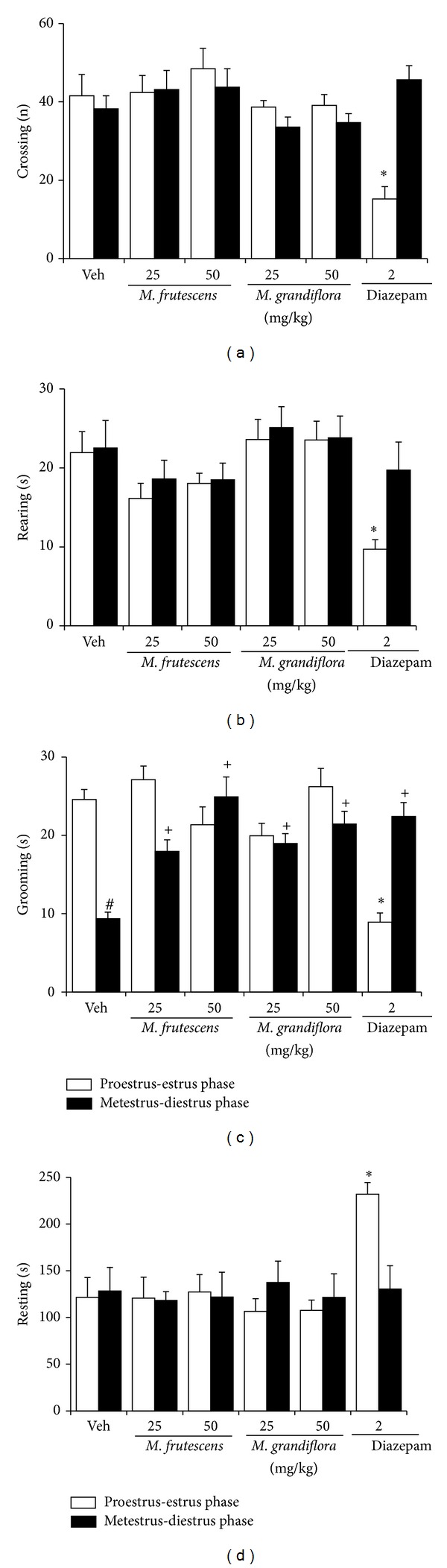
Open field test. (a) The number of crossings, (b) rearing time, (c) grooming time, and (d) resting time during the 5 min test. *P < 0.05, compared with metestrus-diestrus phase in the same group; ^+^ *P* < 0.05, compared with metestrus-diestrus phase in the control group (Veh); ^#^ *P* < 0.05, compared with proestrus-estrus phase in the same group (two-way ANOVA followed by Student-Newman-Keuls* post hoc* test).

The two-way ANOVA did not show statistically significant differences (*F*_1,92_ = 1.07, *P* = 0.303) in the number of crossings between phases of the ovarian cycle, but significant differences (*F*_5,92_ = 4.10, *P* < 0.002) were detected among treatments. The* post hoc* test revealed that diazepam reduced (*P* < 0.05) the number of crossings compared with all of the other treatment groups. The interaction between factors was also significant (*F*_5,92_ = 6.66, *P* < 0.001). The* post hoc* test showed that only rats treated with diazepam during the proestrus-estrus phase exhibited a significant reduction (*P* < 0.05) of the number of crossings compared with all of the other treatment groups in the proestrus-estrus and metestrus-diestrus phases. No other effects of treatment on the number of crossings were detected during the metestrus-diestrus phase (Figure 2(a)).

The analysis of rearing time did not show statistically significant differences (*F*_1,92_ = 3.09, *P* = 0.082) between phases of the ovarian cycle, but significant (*F*_5,92_ = 4.85, *P* < 0.001) differences were detected among treatments. The* post hoc* test revealed that diazepam reduced rearing time compared with all of the other treatment groups (*P* < 0.05). The interaction between factors was not significant (*F*_5,92_ = 1.12, *P* = 0.360). Rats in the proestrus-estrus phase treated with diazepam exhibited a significant reduction (*P* < 0.05) of rearing time compared with all of the other treatment groups in the proestrus-estrus and metestrus-diestrus phases (Figure 2(b)).

The analysis of grooming behavior revealed statistically significant differences (*F*_1,92_ = 4.04, *P* < 0.047) between phases of the ovarian cycle and among treatments (*F*_5,92_ = 7.76, *P* < 0.001) and a significant interaction between factors (*F*_5,92_ = 16.55, *P* < 0.001). The* post hoc* test showed that only rats treated with diazepam during the proestrus-estrus phase exhibited a significant reduction (*P* < 0.05) of grooming time compared with all of the other treatment groups in the proestrus-estrus or metestrus-diestrus phase. No other effects of treatment on grooming behavior were detected during the metestrus-diestrus phase (Figure 2(c)). The analysis of resting time did not reveal statistically significant differences (*F*_1,92_ = 0.66, *P* = 0.416) between phases of the ovarian cycle, but the analysis of this variable revealed statistically significant differences among treatments (*F*_5,92_ = 2.95, *P* = 0.016). The* post hoc* test revealed that diazepam increased resting time compared with all of the other treatment groups (*P* < 0.05). The interaction between factors was also significant (*F*_5,92_ = 2.65, *P* = 0.028). The* post hoc* test showed that only rats in the proestrus-estrus phase treated with diazepam exhibited a significant increase in resting time compared with all of the other treatment groups in the proestrus-estrus or metestrus-diestrus phase (*P* < 0.05). No other effects of treatment on resting time were detected during the metestrus-diestrus phase (Figure 2(d)).

## 4. Discussion

The present study investigated the influence of ovarian cycle phases on anxiety-like behavior in the elevated plus maze in female rats treated with* M. frutescens* and* M. grandiflora* aqueous crude extracts, and the results were compared with the effects of diazepam. The extracts of* M. frutescens* (25 and 50 mg/kg) and* M. grandiflora *(50 mg/kg) produced anxiolytic-like effects similarly to 2 mg/kg diazepam during the metestrus-diestrus phase. The* M. frutescens *and* M. grandiflora* extracts did not exert any behavioral effects during the proestrus-estrus phase, but diazepam produced hypoactivity in the open field test during this phase of the ovarian cycle, reflected by reductions of the number of crossings, rearing time, and grooming time and an increase in resting time. These findings indicate that, similar to diazepam, the* M. frutescens* and* M. grandiflora *extracts produced differential anxiolytic-like effects that depended on the phase of the ovarian cycle but lacked additional anxiolytic-like effects during the proestrus-estrus phase. These data also indicate that a lower dose of the* M. frutescens *extract (25 mg/kg) was required to produce an anxiolytic-like effect compared with the* M. grandiflora* extract (50 mg/kg), suggesting the differential composition of bioactive compounds contained in each plant.

The elevated plus maze is a well-accepted and validated animal model for testing the effectiveness of anxiolytic drugs [23, 26, 33]. In this animal model, rats or mice that display anxiety-like behavior usually show a reduction of open-arm exploration, reflected by a decrease in the time spent in the open arms. Animals treated with clinically effective anxiolytic drugs (e.g., diazepam) [34–36], some neurosteroids with anxiolytic-like potency (e.g., progesterone and allopregnanolone) [37–39], and some extracts from medicinal plants with reputed anxiolytic properties exhibit an increase in the total time spent in the open arms [40, 41], which is considered an anxiolytic-like effect at the experimental level.

The present study found that 25 and 50 mg/kg of the* M. frutescens* extract and 50 mg/kg of the* M. grandiflora* extract decreased anxiety-like behavior in the elevated plus maze in female rats during the metestrus-diestrus phase, when the concentrations of steroid hormones are lower and anxiety-like behavior increases. During the proestrus-estrus phase, when steroid hormones reach higher levels, treatment with the* M. frutescens* or* M. grandiflora* extract did not produce anxiolytic-like effects. The effects exerted by the* M. frutescens* and* M. grandiflora* extracts during the metestrus-diestrus phase were comparable to those observed after diazepam administration, supporting the possible anxiolytic-like profile of the aqueous crude extracts of these* Montanoa* plants.

Similar to diazepam, the* M. frutescens* and* M. grandiflora* extracts did not exert additional anxiolytic-like effects in the elevated plus maze during the proestrus-estrus phase. Only the naturally observed anxiolytic-like effects in this phase of the ovarian cycle (associated with high concentrations of steroid hormones [42]) were observed. These data suggest a “ceiling effect” in the elevated plus maze that prevents the detection of additional anxiolytic-like effects during this phase of the estrous cycle. Other experimental models that evaluate the effects of anxiolytic drugs may detect significant differences in the proestrus-estrus phase. The present results showed that diazepam but not the* M. frutescens* or* M. grandiflora* extract produced hypoactivity in the open field test during the proestrus-estrus phase, reflected by reductions of the number of crossings, rearing time, and grooming time and an increase in resting time. Interestingly, hypoactivity was only identified in the open field test and not in the elevated plus maze, in which the total number of entries into the arms is considered an indicator of motor activity. These results are relevant because they show that the total number of entries into the arms was not an adequate parameter for identifying an effect on spontaneous motor activity. Therefore, the open field test should be specifically used to evaluate motor activity in parallel with the elevated plus maze.

In the present study, the effect on general motor activity may be related to a potential combined effect of diazepam and endogenous steroid hormones (e.g., progesterone or allopregnanolone), which together could act on GABA_A_ receptors and disrupt locomotor activity. This possibility may be supported by previous studies, in which the involvement of GABA_A_ receptors in the anxiolytic-like effects of the aqueous crude extracts of* M. frutescens* [21] and* M. tomentosa* [20] was reported. In those studies, GABA_A_ receptor antagonism blocked the anxiolytic-like effect of the* Montanoa* extracts, suggesting that the GABAergic system mediates the actions of these plants on the central nervous system.

Considering that the proestrus-estrus phase is associated with higher circulating concentrations of steroid hormones recognized by the GABA_A_ receptor [43] and that diazepam effectively targets GABA_A_ receptors, overstimulation of this receptor may have occurred, and female rats may have consequently exhibited hypoactivity in the open field test. This effect on motor activity may be comparable to the effect in the open field test in rats after administration of higher doses of diazepam [21, 44]. This finding suggests that the chemical compounds contained in the aqueous crude extracts of* M. frutescens* and* M. grandiflora* possess low synergistic activity with steroid hormones on the GABA_A_ receptors compared with diazepam to provoke overstimulation of these receptors in central structures that mediate the reduction of anxiety-like behavior. Further studies of synergistic activity are needed to evaluate this possibility.

Grooming behavior increases in rats subjected to mild to moderate stress [45–47], whereas animals subjected to high stress exhibit a significant decrease in this behavior [48, 49] compared with nonstressed animals. The decrease in grooming behavior after high levels of stress can be prevented by anxiolytic drugs, such as diazepam, and other substances with anxiolytic potency, which is considered an additional indicator of anxiolytic-like activity at the experimental level [50–52]. In the present study, the vehicle-treated group showed lower levels of grooming behavior during the metestrus-diestrus phase compared with the proestrus-estrus phase, an effect possibly associated with stress induced by exposure to the elevated plus maze and the reduced concentration of steroid hormones during this phase of the ovarian cycle. A similar pattern of activity in the light/dark test has also been reported in rats with long-term suppression of ovarian hormones [52]. Interestingly, similar to diazepam, 25 and 50 mg/kg of the* M. frutescens* extract and 50 mg/kg of the* M. grandiflora* extract maintained grooming behavior during the metestrus-diestrus phase at a similar level as during the proestrus-estrus phase in vehicle-treated rats. These data support the hypothesis that* M. frutescens *and* M. grandiflora *possess distinctive anxiolytic-like profiles in the elevated plus maze. Additionally, during the proestrus-estrus phase, no effects of the* Montanoa* extracts on grooming and rearing behavior were detected. Nevertheless, diazepam during this same phase of the ovarian cycle reduced both behaviors in the open field test, which may be associated with a relaxation effect of diazepam that is enhanced by steroid hormones during this phase of the ovarian cycle.

One limitation of the present study is that we did not identify the bioactive compounds involved in the anxiolytic-like effects of the extracts or differences in the bioavailability of these bioactive compounds between different plant extracts. Nonetheless, the present results support the traditional use of* M. frutescens* and* M. grandiflora* as potential anxiolytic agents, and further studies should be conducted to identify the chemical compounds and parameters related to their bioavailability that are involved in their anxiolytic-like effects. We can offer a partial explanation of the anxiolytic-like activity exhibited by the extracts analyzed herein by considering phytochemical data on* Montanoa* plants. Pentacyclic triterpenes and tetracyclic diterpenes (e.g., ent-kaurenoic acid, grandiflorenic acid, kauradienoic acid, zoapatanol, and montanol), sesquiterpene lactones, and flavonoids are the main biologically active compounds contained in extracts of* Montanoa* plants [53, 54].* Montanoa* plants and other* Heliantheae* plants contain terpenes with remarkable pleiotropic features. For example, ent-kaurenoic acid possesses both uterotonic and hypoglycemic activity [55]. We propose that the terpenes or flavonoids contained in* Montanoa* plants could be the active compounds involved in the anxiolytic-like effects reported herein by targeting the GABAergic system. Some flavonoids and terpenes with anxiolytic-like activity target GABA_A_ receptors [56–58]. Other compounds, such as sesquiterpene lactones, can also contribute to the beneficial effects of* M. frutescens* and* M. grandiflora* extracts after they enter the nervous system. Thus, sesquiterpene lactones act as alkylating agents, and we have observed antitumoral effects on uterine leiomyomas in women after chronic treatment under specific circumstances (Carro-Juárez et al., in preparation). Toxicological studies are necessary to investigate the safety profile of* Montanoa* extracts to avoid side effects, similar to previous reports for other medicinal plants that act on the central nervous system [59, 60].

In conclusion, the present findings justify further studies that seek to standardize the* M. frutescens* and* M. grandiflora* extracts and identify the bioactive compounds that produce their anxiolytic-like effects. Additionally, the present results may impact the future development of natural therapeutic alternatives to ameliorate symptoms of anxiety in women in whom changes in concentrations of steroid hormones have been reported. The data presented herein further support the traditional use of* M. frutescens* and* M. grandiflora* described by Ximenez in 1615 “*resolves the mood changes and nerves in an admirable form*” [16] for the treatment of “nervous” disorders associated with the menstrual cycle in women.

## Figures and Tables

**Table 1:** Variables evaluated in the elevated plus maze without significant changes associated with treatment and phase of the ovarian cycle.

Group	Number of entries into the open arms		Total number of entries into the arms (open + close)	
	P-E	M-D	P-E	M-D
Vehicle	10.11 ± 1.86	5.63 ± 2.07	13.22 ± 2.08	14.50 ± 2.06
*Mf* (mg/kg)				
25	7.75 ± 1.71	6.50 ± 0.95	13.87 ± 3.56	10.25 ± 2.39
50	7.11 ± 0.91	8.00 ± 1.11	9.56 ± 1.60	10.87 ± 1.79
*Mg* (mg/kg)				
25	5.66 ± 0.67	7.67 ± 0.85	8.44 ± 0.61	9.67 ± 0.57
50	7.11 ± 1.21	7.10 ± 0.96	10.11 ± 0.77	9.50 ± 0.83
Diazepam (mg/kg)				
2	8.78 ± 0.82	8.13 ± 0.97	11.22 ± 1.21	11.25 ± 1.49

No significant differences among subgroups were found, two-way ANOVA. *Mf*: *M. frutescens*; *Mg*: *M. grandiflora*; P-E: proestrus-estrus phase; M-D: metestrus-diestrus phase.
